# Inhibitory effects of curcumin and cyclocurcumin in 1-methyl-4-phenylpyridinium (MPP^+^) induced neurotoxicity in differentiated PC12 cells

**DOI:** 10.1038/s41598-017-17268-3

**Published:** 2017-12-05

**Authors:** Sandeep Chakraborty, Artashes Karmenyan, Jin-Wu Tsai, Arthur Chiou

**Affiliations:** 1 Biophotonics and Molecular Imaging Research Center, National Yang-Ming University, Taipei, 11221 Taiwan, ROC; 2 Department of Life Sciences, National Taiwan University, Taipei, 10617 Taiwan, ROC; 3 Department of Physics, National Dong Hwa University, Hualien, 97401 Taiwan, ROC; 4 Institute of Brain Science, National Yang-Ming University, Taipei, 11221 Taiwan, ROC; 5 Institute of Biophotonics, National Yang-Ming University, Taipei, 11221 Taiwan, ROC

## Abstract

Development and progression of neurodegenerative diseases like Parkinson’s disease (PD) involve multiple pathways. Thus, effective therapeutic treatments should intervene to address all these pathways simultaneously for greater success. Most of the current pharmacotherapeutic approaches just supplement striatal dopamine. Hence, natural extracts of plants with therapeutic potential have been explored. Curcuminoids belong to one such group of polyphenol which show immense therapeutic effects. Here, we have used intracellular reactive oxygen species (ROS) measurement, and two-photon fluorescence lifetime imaging microscopy (2P-FLIM) of cellular autofluorescent co-enzyme reduced nicotinamide adenine dinucleotide (NADH) to study the inhibitory effects of curcumin and cyclocurcumin in alleviating PD like neurotoxicity of 1-methyl-4-phenylpyridinium (MPP^+^) in neuronal growth factor (NGF) induced differentiated PC12 cells. Our results showed that both cyclocurcumin and curcumin reduced the level of ROS caused by MPP^+^ treatment. Moreover, a significant increase in the free, protein-bound, and average NADH fluorescence lifetimes along with a decrease in the relative contribution of free- vs. protein-bound NADH components in curcuminoids treated cells (pretreated with MPP^+^) were observed compared with those treated with MPP^+^ only. This study, which indicates that cyclocurcumin offers higher neuronal protection than curcumin, may initiate further studies of these compounds in the cure of neurodegenerative diseases.

## Introduction

With the increase in average life expectancy around the world, the old age-related neurological disorders pose a real burden for the modern health care system with huge social-economic impact. One such disorder is Parkinson’s disease (PD) which accounts for at least 1% of all the reported neurodegenerative diseases of people aged over 65^1,2^. PD occurs due to the loss of dopaminergic neurons in the midbrain region called *substantia nigra* (SN) causing a dopamine deficiency in the striatum^3,4^. Clinical symptoms of PD include tremor, rigidity, akinesia and problems with balance, in short known as TRAP, while the most important histopathological hallmark is the presence of Lewy bodies in the brain^5,6^. Progression of PD involves a myriad of complicated pathways such as mitochondrial dysfunction, proteasomal inhibition, oxidative stress etc., which have been elucidated to a great extent^3,7^. However, therapeutic procedures for alleviating the progression of PD have met with little success.

Till date, several pharmacological and surgical procedures have been adopted to treat PD^8,9^. Among them, the most frequently used pharmacological treatments are L-dihydroxyphenyl alanine (levodopa, or L-DOPA)^10^, dopamine agonists^11–13^ and monoamine oxidase B inhibitors (MAOB-I)^14,15^. However, all these pharmacological drugs do not protect the remaining functional neurons from degeneration^16,17^. Moreover, the symptomatic reliefs are also temporary. All these facts give rise to the requirement of new pharmacological agents which can protect healthy neurons from degeneration and curative drugs with minimal side effects. Drugs which can protect affected neurons from further degradation either independently or as adjunctive agents with the existing drugs are in demand.

PD is a multifactorial disease^18^, and hence, drugs which can address all these complicated molecular pathways simultaneously for a higher probability of complete cure are required. Hence, effective multi-therapeutic drugs with no side effects and non-toxic to patients have been explored extensively. Among the various possibilities, natural products from plants such as phytochemicals and herbal extracts provide a unique window in the development of multi-therapeutic drugs^19,20^. Most of the documentation of using herbal extracts or phytochemicals in treating diseases can be found in the earlier medicinal texts in countries such as India and China^21^. In recent years, researches to find alternative nature-friendly non-toxic medicines to treat cancer and neurodegenerative diseases have attracted increasing attention.

One such popularly used herb is the rhizome of *turmeric* (*Curcuma Longa*) which has been used in traditional Indian and Chinese medicines for the treatment of various diseases for several centuries and also as dietary spices^21,22^. Curcuminoids represent the family members of the major active group of components of the extracts of turmeric which impart the characteristics yellow color and the medicinal properties^22^. These structurally related polyphenol compounds have been isolated from the roots of turmeric^23^. In traditional medicine, turmeric has been applied to treat asthma and allergy, to enhance the immune system, and to help wound healing (cut, or burn)^24^. Modern day research further revealed a number of other pharmacological activities of turmeric such as anti-microbial, anti-oxidant, and anti-inflammatory^25^. The major constituents of curcuminoids are: curcumin (Curcumin I), demethoxycurcumin (Curcumin II), bisdemethoxycurcumin (Curcumin III), and lately found cyclocurcumin^22^. 77% of commercial curcuminoids consist of curcumin (diferuloymethane, C_21_H_20_O_6_)^23^. From several studies it has been noticed that curcumin has lots of beneficial effects in fighting cancer^26^, cardiovascular disease^27^, obesity^28^, liver disease^29^, and aging^30^. Due to the low toxicity of curcumin and easy availability, it has been studied for potential therapeutic effect against the progression of neurological diseases caused by the formation of aggregated fibrillar protein deposits^31–33^. PD is one such disease and curcumin has been shown to prevent protein aggregation in this debilitating disease^34^. Curcumin as well as demethoxycurcumin, and bisdemethoxycurcumin have been studied widely for medicinal purposes^35^. However, cyclocurcumin, a closely related metabolite which was first isolated from *C*. *Longa* and characterized by Kiuchi *et al*.^36^, has not been studied until recently. Simon *et al*.^37^ have compared the inhibitory effects of curcumin, demethoxycurcumin, bisdemethoxycurcumin, and cyclocurcumin in the cell cycle and cell proliferation of MCF07 human breast cancer cell line. It has been shown that cyclocurcumin inhibits the proliferation of these cells^37^. It has also been suggested that cyclocurcumin may act synergistically with curcumin^36^. In addition to these, inhibition of TNF-α release from lipopolysaccharide (LPS)-stimulated human macrophages following cyclocurcumin treatment has been reported^38^. Thus, to realize the full potential of cyclocurcumin to treat various diseases, the medicinal properties of this compound deserve further studied.

In this work, cyclocurcumin and curcumin have been studied for their inhibitory effects on the neurotoxicity in a cellular model approximating cellular threat conditions in PD. We used 1-methyl-4-phenylpyridinium (MPP^+^), which has been widely used in researches to develop cell and animal PD models^39^, to serve as the active neurotoxin. MPP^+^ can cross the blood-brain barrier and accumulate in mitochondria, and thereby impairing the cellular respiration process^40^. Specifically, PC12 cells (derived from the pheochromocytoma of rat adrenal)^41^ were treated with MPP^+^ to establish the cellular model approximating PD conditions. Hereafter, this cell model will be referred as PD-like cell model. Cell viability, reactive oxygen species (ROS) level, and reduced nicotinamide adenine dinucleotide (NADH) fluorescence lifetime were determined to assess the inhibitory effects of cyclocurcumin and curcumin on the MPP^+^ neurotoxicity. NADH, an auto-fluorescent molecule, is the primary electron donor in the electron transport chain in mitochondria^42,43^. NADH can be found in the cell in either free or protein-bound state. The ratio of free to protein-bound NADH (NADH/NAD^+^), which serves as a cellular metabolic marker^44^, was measured *via* two-photon fluorescence lifetime imaging microscopy (2P-FLIM)^45,46^. Fluorescence lifetime is defined as the average time a fluorophore remains in the excited state before descending to the ground state^47^. It is an intrinsic and inherent property of a fluorophore, independent of the concentration of the fluorophore and its quantum yield^46^, but highly sensitive to the environment, and thus can provide contrast to reveal different cellular microenvironments^48^. This property of fluorescence lifetime was utilized to differentiate and quantify the free and protein-bound NADH, and thereby to monitor cellular metabolism in this study.

## Results

In this work, the inhibitory effects of curcumin and cyclocurcumin on neuro-toxicity in neuronal-like differentiated PC12 cells, caused by MPP^+^ treatment, were evaluated and compared using MTT based cell viability test, intracellular ROS, and NADH fluorescence lifetime measurements.

### MTT cell viability test

The MTT test was performed in NGF-treated differentiated PC12 cells to evaluate the cell viability after different treatments of curcuminoids, and MPP^+^. The viability of the treated cells is presented relative to that of the untreated cells (control) which is regarded as 100% cell viability.

**Figure 1:**
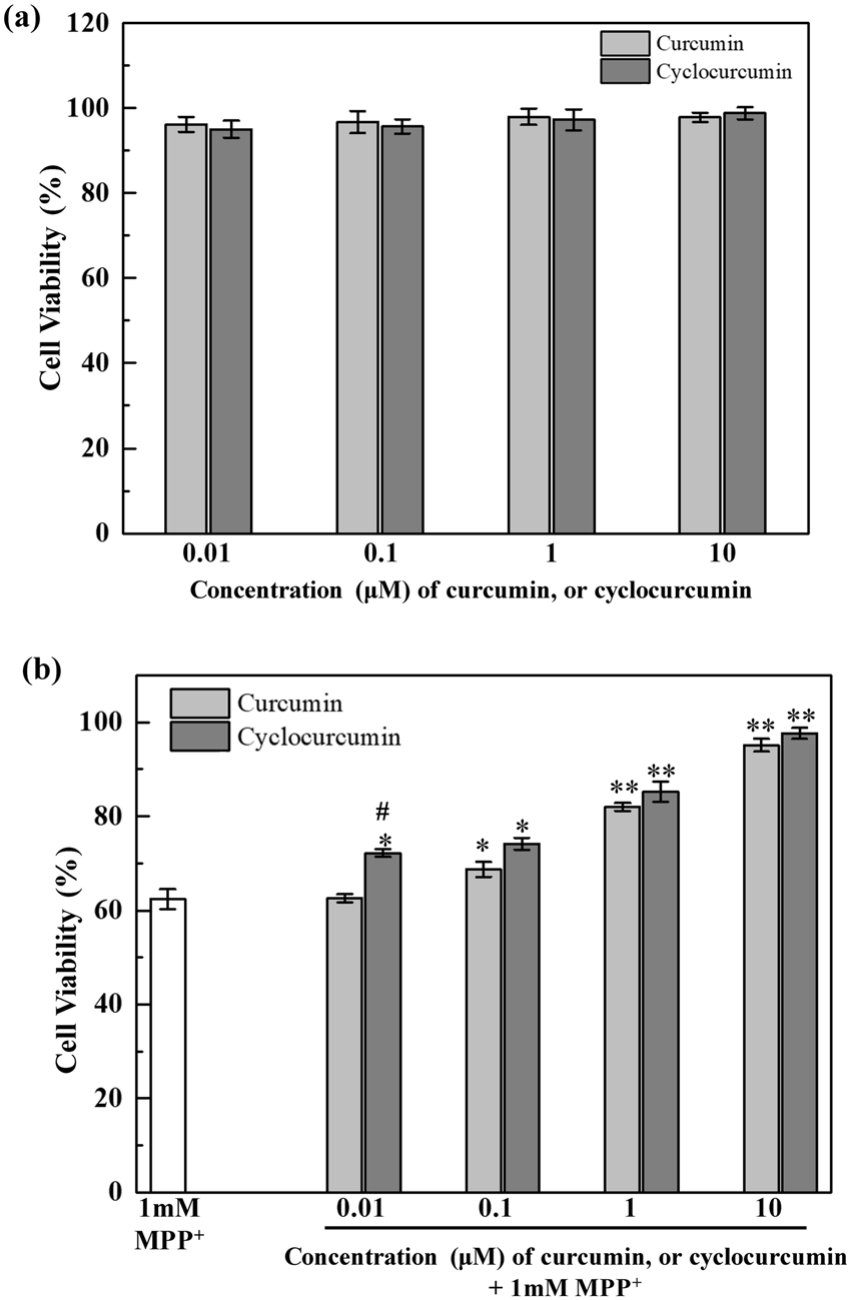
MTT assay of the differentiated PC12 cells. (**a**) Cell viability after the treatments of curcumin, and cyclocurcumin alone (for 24 hours) shows no cytotoxic effects; (**b**) the cell viability increased significantly after the treatment of the combination of curcumin and MPP^+^ in comparison with those treated with MPP^+^ only; the level of recovery is concentration-dependent; Similar results are also observed for cells treated with cyclocurcumin. The data are presented as mean ± standard error of mean (SEM). A one-way ANOVA with LSD post-hoc analysis was performed to specify the statistical significance of the differences between different groups. Statistical significance: **p < 0.001, and *p < 0.05 for 1 mM MPP^+^ treatment alone vs. the combination of curcumin, or cyclocurcumin and MPP^+^ treatments, ^#^p < 0.05 for curcumin vs. cyclocurcumin treatments (LSD test).

Figure 1 summarizes the effect of curcumin and cyclocurcumin in inhibiting the cytotoxic effect of MPP^+^, based on the cell viability of PC12 cells. Our results show that the treatment of curcumin or cyclocurcumin alone (without MPP^+^ treatment) does not affect the viability of the neuronal-like PC12 cells [Fig. 1(a)], inferring that these compounds are not cytotoxic to the differentiated cells within the limits of the concentrations (0.01, 0.1, 1, or 10 µM) used in this study. However, a significant cellular viability reduction (~62%; with p < 0.001) was observed in the differentiated PC12 cells with 1 mM MPP^+^ treatment [Fig. 1(b)]. MPP^+^ treatment induces PD like molecular pathway changes in differentiated PC12 cells, leading to programmed cell death.

Figure 1(b) shows the effect of the combination of curcumin (or cyclocurcumin, 0.01, 0.1, 1, or 10 µM) and MPP^+^ (1 mM) on the viability of the differentiated cells. 0.01 µM curcumin treatment showed no protective effect against MPP^+^ cytotoxicity [Fig. 1(b)]. However, the 0.1, 1, and 10 µM curcumin treatments of the MPP^+^ treated differentiated PC12 cells showed a dose-dependent increase in the viability of the cells. Specifically, cell viability of ~68% (with p < 0.05), 82% (with p < 0.001), and 95% (with p < 0.001) were observed for 0.1, 1, and 10 µM curcumin treatments, respectively, in combination with MPP^+^ treatment, in comparison with the corresponding value (of 62%) for cells treated with MPP^+^ alone. The same set of experiments, repeated with curcumin replaced by cyclocurcumin, yielded similar results [Fig. 1(c)]. Quantitatively, cell viability of 72% (with p < 0.05), 74% (with p < 0.05), 85% (with p < 0.001), and 97% (with p < 0.001) were observed for 0.01, 0.1, 1, and 10 µM cyclocurcumin treatment, respectively, in comparison with the corresponding value (of 62%) for cells treated with MPP^+^ alone [Fig. 1(b)].

### Intracellular ROS measurement

MPP^+^ is an active neurotoxin which can alter the intracellular ROS level in cells. In this work, a ROS sensitive dye H_2_DCF-DA (see “Materials and methods” section) was used to estimate the change in the ROS level in MPP^+^ and curcuminoids treated differentiated PC12 cells.

**Figure 2:**
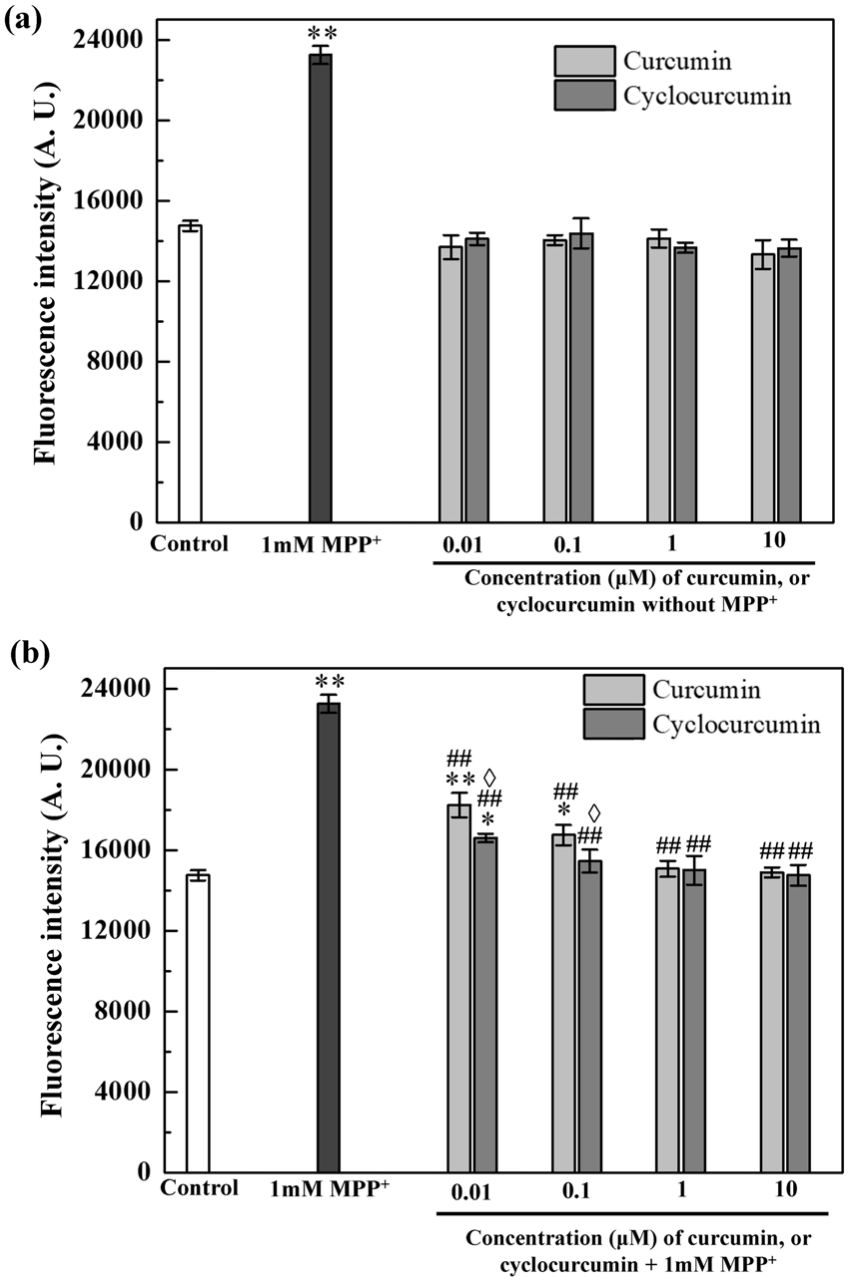
Intracellular ROS levels in the differentiated PC12 cells. (**a**) Intracellular ROS level increased significantly after the MPP^+^ treatment alone; the treatment of either curcumin or cyclocurcumin alone, however, did not change the ROS level of the cells. (**b**) The addition of curcumin, (or cyclocurcumin) after MPP^+^ treatment reduced the ROS level in comparison with MPP^+^ treatment alone. The data are presented as mean ± standard error of mean (SEM). A one-way ANOVA with LSD post-hoc analysis based statistical significance: **p < 0.001, and *p < 0.05 for control vs. cells treated with MPP^+^ or the combination of MPP^+^ and curcumin (or cyclocurcumin); ##p < 0.001 for 1 mM MPP^+^ alone vs. the combination of MPP^+^ and curcumin (or cyclocurcumin) treatments; ^◊^p < 0.05 for the combination of MPP^+^ and curcumin vs. combination of MPP^+^ and cyclocurcumin (LSD test).

Figure 2 summarizes the variation of the intracellular ROS level in the differentiated cells with different chemical treatments. As a control, the effects of curcumin treatment and cyclocurcumin treatment on ROS level were evaluated first. From Fig. 2(a), it can be concluded that the intracellular ROS level did not change after the treatment of either curcumin or cyclocurcumin alone when compared with the control cells. However, after the treatment of 1 mM MPP^+^, a ~36% (with p < 0.001) increase in the intracellular ROS level was observed compared with the control [Fig. 2(a)].

The effect of the combination of curcumin (or cyclocurcumin, 0.01, 0.1, 1, or 10 µM) and 1 mM MPP^+^ on the intracellular ROS level in the neuronal-like cells, as shown in Fig. 2(b), indicates that the increase in ROS level (of ~36% caused by MPP^+^ treatment) was reduced to ~19% (with p < 0.001), and ~11% (with p < 0.05) for 0.01, and 0.1 µM curcumin treatments, respectively, in comparison with the control cells. The corresponding values for 1 and 10 µM curcumin (in combination with MPP^+^) treatments indicated that the recovery was nearly 100% [Fig. 2(b)]. Similar results were observed [Fig. 2(b)] in the case of cyclocurcumin treatments (0.01, 0.1, 1, or 10 µM), except that for the same concentrations (of 0.01 and 0.1 µM) of cyclocurcumin and curcumin, the reduction in ROS level was more effective in the former. For the concentrations of 1 and 10 µM, the recovery was nearly 100% for both.

### NADH fluorescence lifetime measurement *via* 2P-FLIM

**Figure 3:**
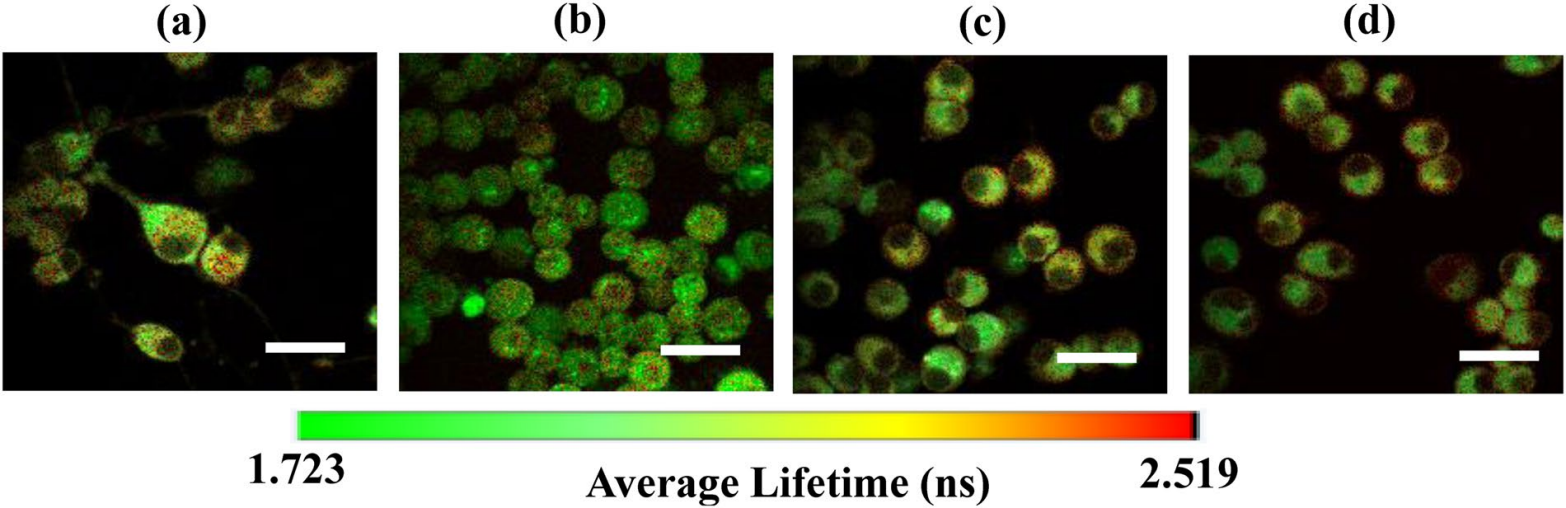
Average NADH fluorescence lifetime images of cells with different chemical treatments. Pseudo-color mapped images of τ_avg_ of differentiated PC12 cells. (**a**) control cells, (**b**) cells treated with1mM MPP^+^ alone, (**c**) combination of 1 mM MPP^+^ and 10 µM curcumin, and (**d**) combination of 1 mM MPP^+^ and 10 µM cyclocurcumin. The color bar shows the distribution of the average NADH fluorescence lifetime (ns) in the cells. Scale bar = 20 μm.

In this study, 2P-FLIM was used for functional mapping of the cellular metabolic state (NADH/NAD^+^) in the differentiated PC12 cells when treated with MPP^+^ alone or in combination with either curcumin or cyclocurcumin. Figure 3 shows the representative average NADH fluorescence lifetime images of the differentiated cells after different treatment conditions. The average NADH fluorescence lifetime (τ_avg_) was calculated as mentioned in “Materials and methods” section. A pseudocolor mapping over the same range was used to represent the distribution of the average fluorescence lifetime in the cells. From Fig. 3(b), it can be seen that the average fluorescence lifetime significantly decreased in 1 mM MPP^+^ treated cells when compared with control [Fig. 3(a)]. Moreover, the average fluorescence lifetime increased after the cells were treated with 10 µM of either curcumin [Fig. 3(c)] or cyclocurcumin [Fig. 3(d)], subsequent to MPP^+^ treatment (of 1 mM). From Fig. 3, it can also be observed that within the same field of view, cell number in the control group differs significantly from the corresponding number of the MPP^+^ treated cells. NGF-treated PC12 cells show neuronal like characteristics and morphology with neuronal varicosities and distribute uniformly in the culture dish; in contrast, after MPP^+^ treatment, the cells tend to aggregate to each other in response to the toxic effect.

**Figure 4:**
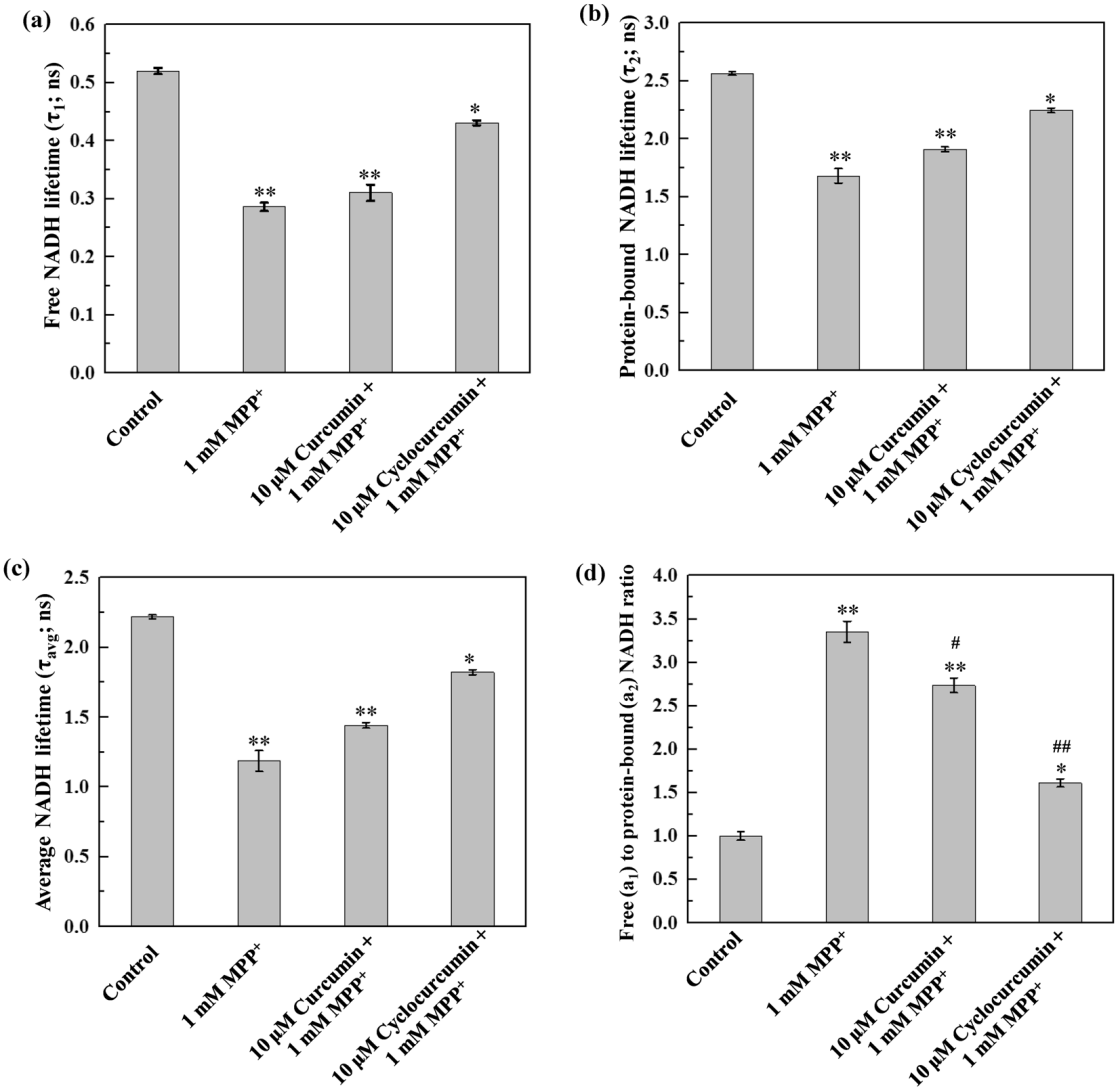
Quantification of the NADH fluorescence lifetime components. (**a**) Free (or short) NADH lifetime (τ_1_; ns) decreased significantly for cells treated with MPP^+^ alone; the decrement in lifetime was partially recovered after subsequent treatment of either curcumin or cyclocurcumin. Similar trends were also observed in (**b**) protein-bound (or long) NADH fluorescence lifetime component (τ_2_; ns), and (**c**) average NADH fluorescence lifetime (τ_avg_; ns). (**d**) The ratio *a*_1_/*a*_2_ increased significantly after 1 mM MPP^+^ treatment alone in comparison with that of the control, but decreased after subsequent treatment of curcumin (or cyclocurcumin). The data are presented as mean ± standard error of mean (SEM). Statistical significance (using a one-way ANOVA with LSD post-hoc analysis). **p < 0.001, *p < 0.05 for control vs. cells treated with MPP^+^ or the combination of MPP^+^ and curcumin (or cyclocurcumin); ##p < 0.001, #p < 0.05 for 1 mM MPP^+^ alone vs. the combination of MPP^+^ and curcumin (or cyclocurcumin) treatments (LSD test).

Further, the NADH fluorescence lifetime components, *viz*., short (or free; τ_1_), long (or protein-bound; τ_2_) and average fluorescence lifetime (τ_avg_), were quantified and shown in Fig. 4, where the averaged values (45 data points) of each NADH lifetime parameters from three independent repetitions on different days are presented. In each set, 15 data points were obtained for each parameter. When compared with the control, τ_1_ decreased to ~45% (with p < 0.001) after 1 mM MPP^+^ treatment. The corresponding values were ~40% (with p < 0.001) and ~17% (with p < 0.05) after subsequent treatment of 10 µM of curcumin and cyclocurcumin, respectively [Fig. 4(a)]. Similarly, τ_2_ decreased to ~34% (with p < 0.001) for 1 mM MPP^+^ treatment only; and the corresponding values were ~25% (with p < 0.001) and ~12% (with p < 0.05) after the treatment of 10 µM of curcumin and cyclocurcumin, respectively, subsequent to 1 mM MPP^+^ treatment [Fig. 4(b)]. Figure 4(c) shows the changes in τ_avg_ for different treatments. It was observed that τ_avg_ decreased to ~46% (with p < 0.001) for MPP^+^ treatment alone; the corresponding values were ~35 (with p < 0.001) and ~17% (with p < 0.05) for cells treated with the combinations of (1) 10 µM curcumin and 1 mM MPP^+^, and (2) 10 µM cyclocurcumin and 1 mM MPP^+^, respectively.

Figure 4(d) shows the ratio of the contributions of free (*a*_1_) and protein-bound (*a*_2_) NADH components, *a*_1_/ *a*_2_, in the cell. This ratio, which is equivalent to NADH/NAD^+^, can be used as a cellular metabolic marker to understand the cellular metabolism^49^. Comparing with the control, *a*_1_/*a*_2_ increased significantly by ~70% (with P < 0.001) after 1 mM of MPP^+^ treatment; while the corresponding values were ~63% (with P < 0.001) and ~37% (with P < 0.001) after subsequent treatment of 10 µM of curcumin and cyclocurcumin, respectively. These results indicate that the amount of intracellular free NADH increased in MPP^+^ treated neuronal-like PC12 cells.

## Discussions

In this study, the neuroprotective effects of curcumin and cyclocurcumin have been studied in PD-like cell model. Randino *et al*. have studied the interaction of cyclocurcumin with Aβ peptides *in vitro* in anionic lipid solution to shed light on how cyclocurcumin can be effective in treating Alzheimer’s disease (AD)^50^. Though the study is interesting, it would have provided more light on the effect of cyclocurcumin in AD if it had been conducted in AD cell model. From this context, our present study can be considered as the first experimental demonstration of cyclocurcumin as potent neuroprotective agent in a cell model approximating a neurodegenerative disease. In addition, in the present PD-like cell model, we used MPP^+^ as a neurotoxin. It has been reported that MPP^+^-induced PC12 cell apoptosis involved higher level of ROS and mitochondrial impairment (which causes a change in the preference of cellular respiration pathway)^18,51^. Based on these earlier results, we have used ROS level and NADH fluorescence lifetime, as the indicator of the change in the cellular respiratory process and of mitochondrial health, respectively, to study the neuroprotective effects of curcumin and cyclocurcumin.

MPP^+^ is a neurotoxin which can significantly reduce the viability of the differentiated PC12 cells. Our experimental results showed that both curcumin and cyclocurcumin can protect the differentiated cells from the cytotoxicity caused by MPP^+^ treatment. The level of rescue is dose-dependent. Moreover, cyclocurcumin treatment turns to be more effective than curcumin, because a significant increase in cell viability was observed for cyclocurcumin treatment with a much lower concentration (of 0.01 µM). To further understand the influence of these treatments, the corresponding changes in the level of intracellular ROS were also studied.

The elevation of the intracellular ROS (oxidative stress) level has been considered as one of the reasons for the MPP^+^ cytotoxicity^52,53^. The intracellular ROS measurement results show that both curcumin and cyclocurcumin alleviated the effect of MPP^+^ on the ROS level in the differentiated PC12 cells, and that the degree of alleviation was dose-dependent. Besides, it can also be seen that, for concentrations of 0.01 and 0.1 mM, the reduction in the ROS level was more effective in the case of cyclocurcumin treatment. These results are consistent with our results in the cell viability assay. Cell viability, among several factors, also depends on the pathway of cellular respiration (oxidative phosphorylation or anaerobic glycolysis), which is strongly influenced by the intracellular ROS level^39,40^. Moreover, as fluorescence lifetime imaging of the cellular respiratory coenzyme NADH can provide useful information about the cellular metabolic state, we conducted the fluorescence lifetime imaging experiments of NADH to quantify the amount of this cellular coenzyme to understand the effect of curcumin and cyclocurcumin on cellular metabolism after MPP^+^ treatment.

MPP^+^ is a potent neurotoxin which severely affects the cellular respiratory process by inhibiting the function of Complex I of the cellular respiratory electron transport chain in mitochondria^39,40^. Our results showed that the protein-bound NADH fluorescence lifetime decreased after MPP^+^ treatment alone. This can be attributed to either the change in its binding sites in Complex I or the change in pH/ROS level in the cells^43^. In our study, we also observed that the ROS level significantly increased in MPP^+^ treated neuronal-like cells. In addition, the decrease in the free-NADH lifetime can be due to the quenching of the nicotinamide moiety by the adenine moiety after MPP^+^ treatment. Our results show that the addition of either curcumin or cyclocurcumin, subsequent to MPP^+^ treatment, may reduce the effect of MPP^+^ on Complex I. The changes in τ_1_, τ_2_, and τ_avg_ were lower after subsequent treatment of either curcumin or cyclocurcumin, when compared with MPP^+^ treatment alone [Fig. 4]. Besides, cyclocurcumin is more effective, in comparison with curcumin, in restoring the values of τ_1_, τ_2_, and τ_avg_ back to their corresponding values in normal condition.

It has been reported that MPP^+^ impairs the transfer of electrons from electron donors to NADH, and thereby increases the amount of free NADH in the cells^53^. The increase in *a*_1_/*a*_2_ ratio also tends to reduce the values of τ_1_, τ_2_, and τ_avg_ [Fig. 4]. Thus, increase in *a*_1_/*a*_2_ ratio indicates a change in the cellular metabolic activity, associated with a shift of cellular respiration towards anaerobic glycolysis from oxidative phosphorylation which is consistent with our previous results^52^. However, when curcumin was used in combination with MPP^+^, the amount of intracellular free NADH was less compared with MPP^+^ treatment alone, as is indicated by the values of *a*_1_/*a*_2_. Moreover, the amount of free NADH was lesser for the combination of cyclocurcumin and MPP^+^ treatment when compared with both MPP^+^ treatment alone and its combination with curcumin. Hence, from the values of *a*_1_/*a*_2,_ it can be concluded that both cyclocurcumin and curcumin could alleviate the effects of MPP^+^ on Complex I and restore the cellular respiration process back to oxidative phosphorylation. From this perspective, we also observed that cyclocurcumin is more effective in protecting the neuronal-like cells from the cytotoxic effect of MPP^+^.

## Conclusions

In this work, we studied and compared the effects of cyclocurcumin with curcumin in inhibiting the cytotoxicity of MPP^+^ in PD cell model. To the best of our knowledge, this is the first reported work of studying the effect of cyclocurcumin in alleviating the oxidative stress in cell model for neurodegenerative diseases. Moreover, the application of fluorescence lifetime measurement in curcuminoids treated cells is also demonstrated here for the first time. The neuronal-like PC12 cells, pretreated with MPP^+^, show higher viability after subsequently treated with cyclocurcumin, or curcumin. Consistent with these results, the intracellular ROS level in the MPP^+^ pretreated PC12 cells was significantly reduced after cyclocurcumin or curcumin treatments. In addition, our results from 2P-FLIM NADH fluorescence lifetime measurements indicate that the change in cellular metabolic activity was restored partially to that of the normal conditions after curcuminoids treatments. The results of these three different approaches correlate very well with each other. The rise in ROS level can significantly reduce the cell viability, while the level of free- to bound- NADH components ratio (*a*_1_/*a*_2_) also bears a positive correlation with the ROS level. In addition, we also present quantitative evidence that cyclocurcumin is more effective than curcumin in neuronal protection in the PD-like cell model, although both deserves further studies for potential application as an alternative drug for treating neurodegenerative diseases.

## Materials and Methods

### Cells preparation

The PC12 cells were maintained in RPMI (Roswell Memorial Park Institute) 1640 medium (Gibco, Life technologies, Massachusetts, USA), supplemented with heat inactivated 10% horse serum, 5% fetal bovine serum (FBS) in a humidified atmosphere in an incubator at 37 °C and 5% carbon dioxide. Cells were grown on laminin/poly-lysine (Sigma-Aldrich, St-Louis, USA) coated 35 mm glass bottom cell culture dishes with a confluence of 1 × 10^5^, and treated with 200ng/ml of nerve growth factor-7S (NGF-7S, derived from murine submaxilliary gland; Sigma-Aldrich, St-Louis, USA) for three days to induce neuronal-like differentiation. Subsequently, the differentiated cells were treated with 1 mM MPP^+^ iodide (Sigma-Aldrich, St-Louis, USA) to develop a PD cell model. To assess the inhibitory effect of cyclocurcumin (ChromaDex, California, USA) and curcumin (Sigma-Aldrich, St-Louis, USA) against the neuro-toxicity caused by MPP^+^, each of them was added separately to the differentiated cells, after two hours of 1 mM MPP^+^ treatment, for 24 hours. The concentration of 1 mM MPP^+^ was chosen to reduce the viability of the cells to around ~60%; besides, our previous study shows that this concentration is appropriate to induce PD like metabolic changes in the cells^49^. We then determined (i) the cell viability, (ii) the intracellular ROS level, and (iii) the NADH fluorescence lifetime, using 3-(4, 5-dimethylthiazole-2)-2, 5-diphenyltetrazolium bromide (MTT) assay, redox-sensitive dye carboxy 2′7′-dichlorodihydrofluorescein diacetate (H_2_DCF-DA), and two-photon fluorescence lifetime imaging microscopy (2P-FLIM), respectively, of four types of cells samples, namely, (1) without any treatment (control), (2) treated with 1 mM MPP^+^ only (for 24 hours), (3) treated with 1 mM MPP^+^ (for 2 hours) followed by curcumin (for 24 hours), and (4) treated with 1 mM MPP^+^ (for 2 hours) followed by cyclocurcumin (for 24 hours). During the acquisition of 2P-FLIM images, the cells were transferred to a loading buffer containing 10 mM HEPES, 2.2 mM CaCl_2_.H_2_O, 150 mM NaCl, 5 mM KCl, 5 mM MgCl_2_.H_2_O, 5 mM glucose with pH 7.4, Osm 300 to maintain physiological conditions. Moreover, the microscope stage was equipped with a temperature control to maintain the cells at 37 °C.

### MTT assay for cell viability measurement

MTT assay is a colorimetric based assay which has been widely used to study cell viability and cell proliferation under different external factors^54^. Metabolically active cells selectively reduce yellow tetrazolium salt MTT (Sigma-Aldrich, St-Louis, USA) into purple-blue insoluble formazon crystals, which can be quantified using spectroscopic measurements. In this study, the MTT assay was used in three replicates in 96 well black with clear flat bottom dishes (R&D Systems, Minneapolis, USA). An MTT stock solution of 5 mg/ml in phosphate buffer saline was prepared. After incubation of the cells with MPP^+^, curcumin, and cyclocurcumin, as was described above (under “Cells preparation” sub-section), the treatment medium was removed and the cells were further treated with serum free medium containing MTT (10 µl per 100 µl) for 4 hours at 37 °C. Subsequently, the MTT solution was discarded and 200 µl of dimethoxy sulfoxide (DMSO) was added to each well to solubilize the formazon precipitate. The extraction procedure was performed for 10 minutes at 37 °C. The amount of formazon was estimated by measuring the optical density (OD) at 570 nm with a reference wavelength of 670 nm in a fluorescence multiplate reader (Tecan Infinite M200 pro, Männedorf, Switzerland).

### Reactive oxygen species (ROS) measurement

The intracellular ROS level was quantified using a redox-sensitive dye carboxy 2′7′-dichlorodihydrofluorescein diacetate (H_2_DCF-DA) obtained from Molecular Probes, Oregon, USA. H_2_DCF-DA is non-fluorescent; however, upon oxidation by ROS it converts to fluorescent marker 2′7′-dichlorofluorescein (DCF) which emits green fluorescence. Prior to use, a stock solution of 100 mM H_2_DCF-DA in DMSO was prepared. The PC12 cells with MPP^+^, curcumin, and cyclocurcumin treatments were prepared, as was described above (under “Cells preparation” sub-section). For ROS measurements, the control and the treated cells were washed with PBS to remove the original medium. Subsequently, the cells were treated with 1 μM of H_2_DCF-DA (reconstituted in RPMI medium from the stock solution) for 45 minutes in a conventional incubator (37 °C, 5% CO_2_). The cells were then washed thrice with PBS to remove the additional dyes and re-suspended in the original cell medium for measurements. DCF fluorescence intensity was measured using a fluorescence multiplate reader (Tecan Infinite M200 pro, Männedorf, Switzerland) with 488 nm excitation and 538 nm emission filters.

### 2P-FLIM instrumentation and fluorescence lifetime measurement

The details of 2P-FLIM instrumentation and fluorescence lifetime measurement can be found elsewhere^49^. Briefly, here, a Ti: sapphire laser (Tsunami, Spectra-Physics, USA) source producing picosecond laser pulses with 82 MHz repetition rate in the spectral range of 740 to 920 nm was used for two-photon excitation. For NADH fluorescence lifetime imaging, the central wavelength of the laser was set at 760 nm for excitation. A 447/60 nm band pass filter (Semrock, New York, USA) was used in the detection arm to select the NADH autofluorescence emission signal. The experiments were performed *via* an inverted Olympus IX71 microscope. A 60x, 1.2 numerical aperture (NA), water immersion plan apochromatic microscope objective (UPlanSApo, Olympus, Japan) was used to focus the laser beam on the sample. The average laser power (exiting the objective lens) was maintained at 4 to 5 mW. All the NADH FLIM images were recorded with 256 × 256 pixels, and the image acquisition time was ~700 secs per image to acquire sufficient photon counts for subsequent data analysis.

The fluorescence lifetime was determined using a time domain technique wherein the time difference between the excitation and the corresponding emission photons were recorded using a time-correlated single photon counting (TCSPC) electronic module (PicoHarp 300, PicoQuant GmbH, Germany). A photomultiplier tube (PMC100, Becker & Hickl GmbH, Germany) was used to detect the NADH fluorescence emission signal. This fluorescence emission signal and the reference laser excitation signal (detected by a Photodiode, TDA 200, PicoQuant GmbH, Germany) were coupled to a photon counting electronic module (PicoHarp 300). TCSPC works in a reverse START-STOP mode where the START signal is triggered by the emission signal, and the STOP signal by the reference laser signal. TCSPC is generally operated in a time-tagged time-resolved (TTTR) mode to resolve the decay curve.

The FLIM data as obtained above were analyzed using a commercially available software package (SymPho Time, version 5.2.4.0, PicoQuant GmbH, Germany). A curve-fitting of a decay model function with the experimental decay data is required to obtain physically meaningful information. As a practice, the decay model function was first convolved with the instrument response function (IRF) of the system, and subsequently fitted with the experimental decay data. However, to further take into account the presence of background noise from the detector, and (or) the temporal shift between the IRF and the decay, a non-linear least square fitting procedure was adopted instead of direct fitting. In this work, the IRF was calculated from the second harmonic generation (SHG) of a urea thin film; the FWHM of the IRF was determined to be 181 ps. A double exponential decay model function, $f(t)={a}_{1}{e}^{-t/{\tau }_{1}}+{a}_{2}{e}^{-t/{\tau }_{2}}$ was used to fit the NADH fluorescence decay curve. Here, *τ*_*1*_ and *τ*_*2*_ denote the short (or free) and long (or protein-bound) fluorescence lifetime components of NADH, whereas *a*_1_ and *a*_2_ denote the corresponding contributions of these components, respectively. The *a*_1_/*a*_2_ ratio is used to quantify the relative contributions of free vs. protein-bound NADH (NADH/NAD^+^). The average NADH fluorescence lifetime was calculated from ${\tau }_{{avg}}=({a}_{1}{\tau }_{1}+{a}_{2}{\tau }_{2})/({a}_{1}+{a}_{2})$. The NADH FLIM data were analyzed pixel-by-pixel, with signal higher than 1000 counts per pixel after binning.

### Statistical analysis

One-way analysis of variance (ANOVA) with least square difference (LSD) was used to determine statistically the significance of the differences between different groups of data. The null hypothesis was rejected for p values lower than 0.05. The LSD test is generally applied after the null hypothesis is rejected in one-way ANOVA and to compare the experiment sets taking into account the pooled standard deviation from all the groups. ANOVA analysis was performed after verifying the normal distribution of the data set. The data are presented as mean ± standard error of mean (SEM).
